# TRIPS, pharmaceutical patents, and generic competition in India

**DOI:** 10.1093/haschl/qxaf239

**Published:** 2025-12-17

**Authors:** Margaret K Kyle, Bhaven N Sampat, Kenneth C Shadlen

**Affiliations:** Center for Industrial Economics, Mines Paris - PSL, Paris 75005, France; School of Government and Policy, Johns Hopkins University, 555 Pennsylvania Avenue NW, Washington, D.C. 20001, United States; Department of International Development, London School of Economics and Political Science (LSE), London WC2A 2AE, United Kingdom

**Keywords:** pharmaceutical patents, TRIPS, generics, competition, India

## Abstract

**Introduction:**

India introduced pharmaceutical patents in response to the 1995 Trade-Related Aspects of Intellectual Property Rights (TRIPS) Agreement. Previous studies that focused on older drugs showed limited effects of patents on India's generic market. We examine how a transitional TRIPS provision, which made patents with first global filing (priority) before 1995 ineligible, affected the likelihood of drugs obtaining strong “primary” patents in India, and the subsequent effects on generic competition.

**Methods:**

We determined the primary patent priority year (PPPYear) for drugs approved in the United States 1995-2017. For each drug, we gathered Indian patent information and recorded the level of generic competition five years from launch.

**Results:**

Primary patents are much more common after PPPYear 1995. Indian generic competition falls approximately 40% after this cutoff, especially for drugs with Indian primary patents.

**Conclusion:**

Post-1995 PPPYear drugs are more likely to have primary patents and face less generic competition in India. These drugs now constitute most approvals in India and better reflect the long-run impact of TRIPS than drugs previously examined. The prevalence of drugs in India with primary patents and less competition has important implications for global access to medicines.

## Introduction

The World Trade Organization's 1995 Agreement on Trade-Related Aspects of Intellectual Property Rights (TRIPS) required developing countries to change their patent laws to make them similar to those in the United States, Europe, and Japan, including the granting of pharmaceutical product patents. Because patents have the potential to limit competition and increase prices, the global extension of pharmaceutical patents generated concerns about reduced access to essential medicines, particularly in poorer countries with constrained health budgets and patients' drug purchases often made out of pocket.^1-3^ Indeed, it is possible that this global change could exacerbate global inequalities around access to medicines,^4^ inequalities that were brought into sharp relief by the distribution of essential medical countermeasures during the COVID-19 pandemic.^5,6^

These concerns have been particularly acute in the case of India. Beginning in the 1970s, with pharmaceutical patent protection limited to manufacturing processes, a sophisticated national industry emerged, skilled at reverse-engineering drugs developed by multinational firms. Indian companies became suppliers of inexpensive medicines, for local and global markets, and the country's pharmaceutical sector became known in public health circles as “the pharmacy to the developing world.”^7,8^

Early post-TRIPS studies on India found limited effects on competition or prices,^9,10^ however, suggesting these concerns may have been misguided. Beyond India, a study of access to WHO-listed “essential medicines” in 65 low and middle-income countries also dismissed the effects of patents.^11^

We go beyond previous research by using more up-to-date data and by exploiting two important characteristics of this setting. First, we call attention to patent heterogeneity, distinguishing between “primary” patents, which cover new drug molecules, and “secondary” patents, which cover alternative forms and presentations of existing molecules^12-15^ Primary patents have stronger blocking effects because they have clearer legal boundaries, are harder to invent around without infringing, and, when litigated, are more likely to be upheld as valid than secondary patents.^16,17^

Second, we focus on the timing of eligibility for primary patents in India. TRIPS required countries to allow patents on pharmaceutical products starting in 1995, when the agreement came into effect; the new rules did not apply to inventions with earlier, pre-1995 “priority” (first global filing) dates. In countries like India that adhered strictly to this cutoff (pre-1995 priority: ineligible; post-1995 priority: eligible), the lags between patent filing and drug launch that are intrinsic to the pharmaceutical industry meant that more than a decade would pass before the majority of drugs launched after TRIPS came into effect could receive primary patents.

Our focus on the set of drugs fully “treated” by TRIPS, specifically those whose primary patents have priority dates of 1995 or later and thus were eligible for protection in India, allows us to go beyond previous work and provide evidence on the steady-state impact of this historic international agreement.

## TRIPS and India: the importance of the 1995 priority cutoff

Despite the widespread concerns that TRIPS would reduce access to medicines, early empirical research on realized outcomes in India did not identify substantial effects on pharmaceutical competition and drug prices. One analysis looks at the market status in India of 184 molecules that were introduced in the United States between 2000 and 2009.^9^ The authors find high rates of generic competition, even though all the drugs in their sample were launched after TRIPS. Another study examines about 1000 molecules that were on the Indian market in 2005, when India started granting pharmaceutical product patents, observing these until 2011.^10^ Using “within-molecule” changes in patent status over time (specifically, the addition of patents to a molecule), the authors find negligible effects of patents on competition and small (but statistically significant) effects on prices.

These findings could, in theory, be driven by the way that India implemented TRIPS, including the adoption of several measures designed to minimize the effects of the introduction of pharmaceutical patents.^18-20^  Appendix 1 reviews aspects of TRIPS implementation in India and discusses the possible relationship between India's pharmaceutical patent regime and the findings of the previous literature referenced above.

Rather than focus on the specifics of India's pharmaceutical patent system, our analysis examines the impact of a first-order characteristic of TRIPS that has not received adequate attention in previous studies: countries were not expected to acknowledge patents filed before 1995. Accordingly, only patents with priority dates of 1995 and onward were eligible in India. Drugs with primary patents that have earlier priority dates only qualified for secondary patents filed after 1995 and, as explained above, secondary patents may be easier to invent around and more susceptible to invalidity challenges. In short, the key factor in determining whether a drug is likely to benefit from strong patent protection in India is whether the priority date of its *primary* patent is before or after 1995. In the remainder of the text we refer to this date as the primary patent priority year, PPPYear.

## Data and methods

We start with a dataset of drugs approved in the United States since 1995. We then collect the patent information on these drugs, their launch dates in India, and the extent of unauthorized Indian generic competition for these drugs.

*New molecular entities*: We start with the set of drugs approved in the United States between 1995 and 2017. We use data from the 2018 version of the Drugs@FDA database on all new molecular entities (NMEs) approved in this period.^21^ In our analyses we use the US Food and Drug Adminstration's (FDA) approval date as the proxy for global launch.

*Orange Book patent data*: Since we are interested in patented drugs, we further restrict the set to NMEs approved over this period with at least one U.S. patent listed in the FDA's Orange Book, using the NBER dataset covering 1985-2016 editions^22^ and the online version.^23^ The sample includes 550 distinct molecules.

*India patent data*: Information on all Indian patents and applications for the 550 NMEs comes from IQVIA's Ark Patent Intelligence Database.^24^ We verified the accuracy of Ark against previous approaches to manually landscape Indian filings,^25^ finding in almost all cases that Ark included the relevant patent filings. We also compared Ark's U.S. patent landscapes to FDA Orange Book data, again confirming comprehensiveness.

*Patent codings*: Ark classifies patents by category and subcategory (Appendix 2). Based on the descriptions in Ark's documentation, and comparison with expert coding described in previous research,^15,16^ we determined that the subcategory “Molecule patent” corresponds most closely to our definition of primary patents. We refer to the “Molecule Patent” subcategory as “primary,” and all other patent types as “secondary.” Since we focus on the differential effect of primary and secondary product patents on competition, we drop any Indian patents Ark categorized as process patents (which were available before TRIPS). For the United States, we rely on Ark codings of patents also listed in the Orange Book (which excludes process patents).

*PPPYear*: Ark also provides data on the priority year of each patent, the first global filing date. We define a drug's PPPYear, the priority year of its primary patent, based on the U.S. data. For purposes of these calculations, we focus on the 448 drugs (out of 550) with a primary patent in the United States.

In principle, the number of drugs with PPPYear before 1995 with primary patents in India should be zero, if Indian patent examiners were perfectly applying the law and if IQVIA correctly coded the patents. In reviewing the small number of pre-1995 PPPYear drugs that are recorded as having primary patents in India, we found a few of these to be secondary product patents or process patents. Despite this noise, overall we have confidence in Ark patent coding. Hemphill and Sampat^17^ validate Ark coding vs. manual expert coding of U.S. patents, and there is no reason to believe the accuracy of Ark coding is any different for Indian patents (written in English, with similar structure to U.S. patents).

*Market data*: To obtain information on market competition, we use IQVIA's MIDAS dataset,^26^ which provides information on the dates that all products containing specific molecules were first sold in given markets. We construct the number of producers for a given drug at specific dates. Molecules have multiple forms (e.g. tablet vs. suspension; different dosages). We focus on the molecule level, considering the number of producers of any form of that molecule. This is our preferred unit of analysis because primary patents are at the molecule level, but we obtain similar results if we estimate entry into each form.

*Market competition in India*: Of the 448 NMEs with primary patents in the United States (for which we could assign a PPPYear), 260 mapped to products sold in India. Our main outcome variable is “generic” competition. We distinguish between licensed generics (produced with the permission of the patent holder) and independent generics, and focus on the presence of the latter. We classify any producer that MIDAS categorizes as an “Other Brand” product (as opposed to “Original Brand” or “Licensed Brand”) as an independent generic in India. Since license agreements are not public, and it is possible that some of the producers we classify as independent generics are in fact licensed but not tracked by MIDAS, we consider this measure an upper bound of our outcome variable. (We also note that the term “generic” has different meanings in different settings. In the United States, “generic” drugs are approved via a regulatory pathway that requires a demonstration of bioequivalence to a reference drug and are typically commercialized using an international non-proprietary name. In India, and many LMICs, “generic” drugs are not necessarily bioequivalent and are commonly sold with brand names.)

## Results

Using these data, we examine how drug approvals, Indian patenting and generic competition vary by PPPYear. Appendix 3 provides a data overview diagram that explains which drugs are used in each step in the analysis.

### Drug approvals by PPPYear

Though most of our empirical analyses focus on the subset of products launched in India, we first examine the relationship between PPPYear and global launch year of the full set of 448 NMEs, to illustrate that only recently approved drugs are fully covered by TRIPS.

**Figure 1.**
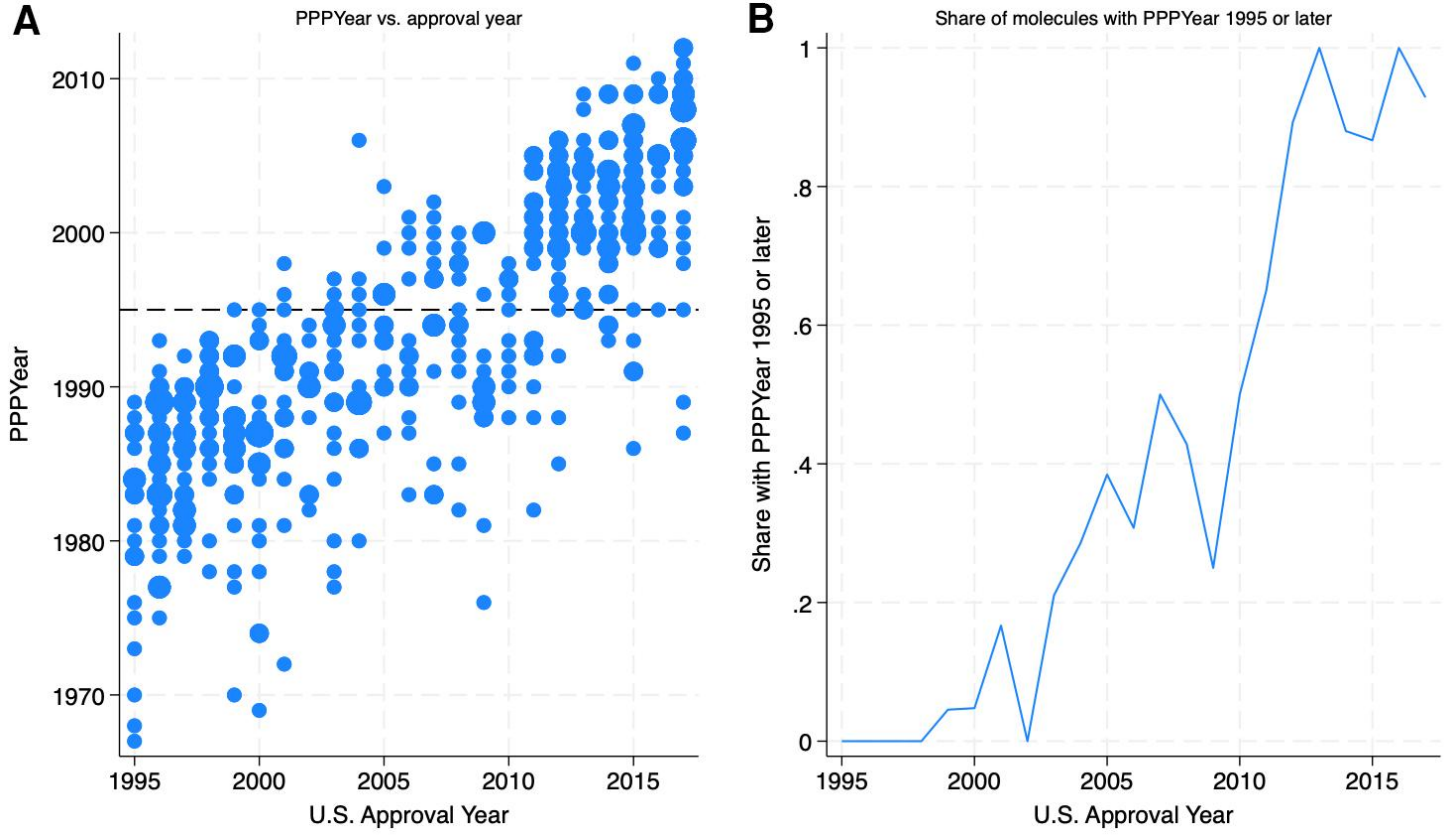
U.S. Approval Year and PPPYear. Sources: Authors' analysis of FDA Orange Book and IQVIA Ark data.

The first panel of Figure 1, which extends earlier work^27^ with more precise measures of drug-level priority years and over a longer period of time, shows the approval year in the United States on the horizontal axis, and PPPYear on the vertical axis. The reference line is at priority year 1995, and each bubble is scaled to the number of drugs with a given priority year-approval year. Among drugs approved since 1995, most have primary patents with pre-1995 priority until about 2010. The second panel displays these data in terms of the share of drugs approved in each year that have PPPYear of 1995 or later. As explained, for countries introducing pharmaceutical patent systems following TRIPS, the requirement to acknowledge patent applications starts in 1995; strict adherence to this provision meant that only post-1995 PPPYear molecules would be eligible for strong, primary patent protection.

The relationship between approval date and PPPYear is important for understanding the Indian case, because previous analyses used samples with earlier drugs. Berndt and Cockburn^9^ focus on drugs approved globally between 2000 and 2010, and Duggan et al.^10^ study drugs on the Indian market between 2003 and 2011. During both periods, most drugs had earlier, pre-1995 PPPYears and were thus ineligible for strong, primary patent protection in India.

### Indian patenting by PPPYear

**Figure 2.**
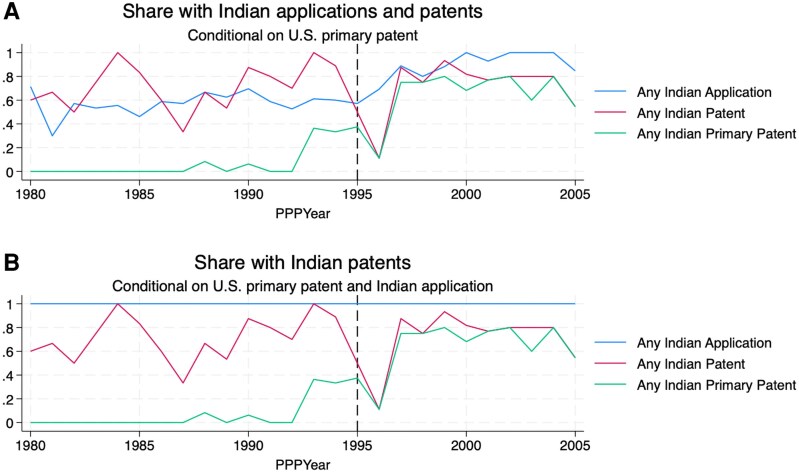
Share with Indian Applications and Patents. Sources: Authors' analysis of FDA Orange Book and IQVIA Ark data.

To test the relationship between PPPYear and Indian patenting, we focus on the 385 drugs with PPPYears from 1980 to 2005. The first panel of Figure 2 shows the share of drugs with an Indian application, any patent, or a primary patent, by PPPYear. The second panel shows the patent information conditional on the drug having an application filed in India.

We observe continuous increases in the shares of drugs with applications and granted patents by PPPYear. By the end of the period, almost all drugs have an Indian application, and most have at least one patent. Even pre-1995 PPPYear molecules can get patents in India, typically secondary patents with post-1995 filing dates. The dashed line suggests no obvious break in trend for patents overall.

Primary patents, however, are rare for drugs with PPPYear before 1995, increasing sharply afterward. The majority of post-95 PPPYear drugs not only have a patent, but a primary patent. Similar results are seen in the second panel, focusing on the subset of drugs with applications filed in India. While there is no sharp break around PPPYear 1995 for patenting overall, there is for primary patents.

### Indian competition by PPPYear

Table 1 summarizes the differences in generic competition between pre-1995 and post-1995 PPPYear drugs, by patent type. Overall, the average number of independent producers is lower for drugs with primary patents (1.29) than it is for drugs with secondary patents only (6.55) and drugs with no product patents (5.33). And, importantly, drugs with primary patents become much more common after PPPYear 1995.

**Table 1.** Pre-1995 vs Post-1995 PPPYear comparison

	PPPYear		
	Pre-1995	Post-1995	Total
**Indian patent status (year 5)**			
No product patent			
Mean	5.38	5.18	5.33
Number of non-missing values	112	34	146
Primary product patent			
Mean	1.33	1.29	1.29
Number of non-missing values	3	35	38
Secondary product patent only			
Mean	6.64	5.89	6.55
Number of non-missing values	67	9	76
Total			
Mean	5.77	3.51	5.10
Number of non-missing values	182	78	260

Authors' analysis of Ark and MIDAS data.

**Figure 3.**
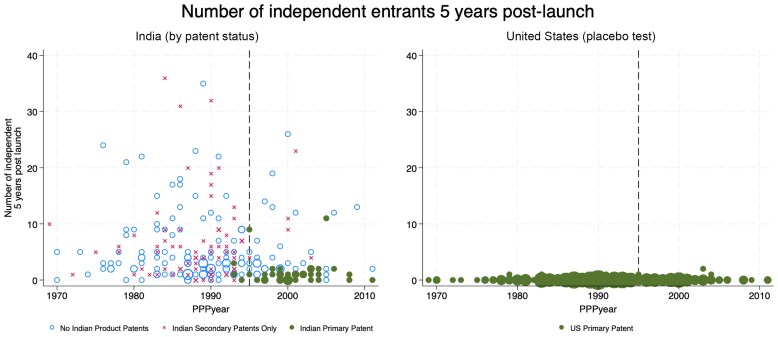
Independent Entry. Sources: Authors' analysis of IQVIA Ark and IQVIA MIDAS data. *Note:* Marker size is proportional to the number of drugs at each point. India panel shows three groups by patent status. All drugs in the U.S. panel have primary patents, by construction.

Figure 3 displays our main results. The first panel shows how entry by independent firms five years after launch varies by PPPYear for drugs with and without primary patents in India. The markers are scaled to reflect the number of drugs in the data at each year. The number of independent producers drops by about 40% after PPPYear 1995 (see Tables A.9 and A.10 in Appendix 4 for further details). This is most pronounced for drugs with primary patents in India, which are also more common after PPPYear 1995. The second panel, which shows equivalent data for the United States, serves as a placebo test. In the United States, where product patents were available before TRIPS, we see no obvious break in trend for the same drugs at PPPYear 1995. (Recall that all drugs in the sample have U.S. primary patents, by construction.)

We also examined the relationship between primary vs secondary patents and generic competition in a regression framework, controlling for drug characteristics that could influence both patents and generic entry. Drugs with unexpired Indian primary patents 5 years after launch have a more than 30% point lower likelihood of generic entry than unpatented drugs. Secondary patents are also associated with reduced generic entry, but the magnitudes are smaller and statistically insignificant. The results are robust to various other approaches and measures. Details are provided in Appendix 4.

## Discussion

The 1995 TRIPS Agreement was among the largest changes in the history of global patent policies. In this paper, we provide new evidence on its effects in the sector and country where this policy change was most controversial: the pharmaceutical industry in India. In contrast to early studies on TRIPS in India, based on older drugs, we find substantially lower generic competition for drugs fully covered by the new rules.

Our analyses highlight the importance of a key dimension of TRIPS that has not received adequate attention: the 1995 priority year cutoff for patent eligibility. We show the interactions between priority dates of patents, patent types, the strength of patent coverage, and degrees of generic competition. The analyses reveal a shock to primary patents and competition in India for drugs that were eligible for strong patent protection.

An important takeaway is that international agreements that include transition periods for national implementation can take time to have effects. This general feature of international agreements is particularly crucial for understanding the effects of TRIPS on pharmaceutical markets, given the transition periods for national implementation, the exemption of patent applications with pre-1995 priority dates, and lags between priority year and launch dates. Drugs with post-1995 priority year for their primary patents represent the steady-state impact of TRIPS better than the sample of drugs examined in previous studies.

To be sure, the relevance of the 1995 cutoff date was acknowledged, if not emphasized, in previous work. In an endnote, Berndt and Cockburn^9(note 23)^ explain that “as the proportion of new drugs that are ‘grandfathered out’ of eligibility for patent protection by being invented too early falls,” the dynamics of genericization may differ from what they found. Duggan et al.^10^ also recognize that the effects of TRIPS are likely to be greater for more recent drugs. In their analyses, however, rather than distinguish drugs according to whether the primary patent priority year was before or after 1995, Duggan et al. consider drugs launched after 1995 in India to be covered by TRIPS. The authors acknowledge that this is an imperfect measure, referring to launch date as the “upper bound” of the priority year. As we have shown in Figure 1, the gaps between priority and launch dates can be substantial. Sure enough, when we study drugs with post-1995 PPPYear, we observe both an increase in the share with primary patents and a concomitant reduction in generic competition. Indeed, the two panels of Figure 3 suggest that the Indian market resembles the U.S. market since PPPYear 1995, reflecting the prevalence of drugs that are fully subject to TRIPS and have primary patents, and the limited degree of competition faced by drugs with primary patents.

While we believe our results are more representative of the long-run steady-state effects of TRIPS than previous work focused on older molecules, there are several limitations. First, we do not code patents directly as primary or secondary, instead relying on the coding done by Ark. This coding, like manual coding, can be subject to noise, and relies on judgement calls. While we did not cross-validate Ark coding specifically for this paper, previous work^17^ did so for U.S. patents on drugs with first generic entry between 2001 and 2010, finding that for 95% of patents, Ark coding agreed with independent expert coding. The extent of misclassification in Ark coding is small, and unlikely to affect our analyses. In any case, to the extent there is misclassification of primary patents as secondary (or process), this would bias the results toward no effects, suggesting that our results provide a lower-bound of the importance of primary patents.

Second, we did not focus on prices, but rather competition, on the grounds that competition is the key channel through which patents should affect price. We expect that our finding that drugs with post-1995 PPPYear (and thus eligible for primary patents in India) are the ones which matter for competition, would extend to prices, but did not examine this directly.

The third limitation is that our analyses are cross-sectional. While we control for relevant factors in the regression models, it is possible that unobserved features of drugs may affect both patent protection and competition. We do not undertake within-molecule analysis of the effects of TRIPS on competition, tracking the effects of a drug being switched from “unpatented” status to “patented,” which would absorb some of the unobserved heterogeneity. The reason why is that very few (if any) drugs receive primary patents after launch, so such an analysis could only identify the effects secondary patents. To estimate the effects of primary patents on competition (or prices) in India, one could instead examine within-molecule dynamics before and after primary patent expiration. This is how the effects of patents are typically identified in the U.S. market. While we did not have sufficient data to explore this for this paper, we intend to explore this in future work.

Notwithstanding these limitations, our analyses provide broader lessons for research on patents and access to medicines. Just as in the United States, assessments of the effects of patents on outcomes must account for heterogeneity in patent type and strength. Three decades into the TRIPS era, the distinction between patent types is crucial for understanding challenges around global access to medicines.

Another lesson is that, for assessments of the national impacts of the globalization of pharmaceutical patenting, specifics around timing matter for isolating which drugs are likely to receive strong patent protection. While India did not grant pharmaceutical patents until required to do so by TRIPS, and in doing so adhered strictly to the 1995 cutoff, other countries adopted different approaches. The post-apartheid government in South Africa inherited a pharmaceutical patent regime, for example, and Mexico and Thailand began granting pharmaceutical patents in the early 1990s. In each of these countries, many drugs with PPPYear before 1995 were eligible for primary patents because this transitional provision of TRIPS did not apply. Or consider Brazil, which (like India) did not introduce a pharmaceutical patent regime until required by TRIPS, but did so with a “pipeline” mechanism that allowed even drugs with earlier pre-1995 priority to obtain primary patents; in Brazil, many drugs that were too old to be eligible for strong patent protection in countries like India did receive primary patents.^25,28^ In general, across the globe, there is considerable variation in what subset of drugs qualify for primary patent protection and thus should be considered “treated” or “untreated” by TRIPS.

The policy implications of our research for access to medicines extend beyond India. As countries attempt to negotiate lower prices with the providers of patented drugs, they sometimes threaten to issue compulsory licenses, which expose the patent-holder to competition. Importantly, the usefulness of such threats depends on the existence of alternative suppliers to the patent-holder, if not locally then globally. Indian pharmaceutical firms have regularly provided this alternative source of supply, able to do so on account of the country's adherence to the 1995 deadline and the lack of primary patents on pre-1995 drugs. Yet as more drugs enter the Indian market protected by primary patents, and therefore single-source products until the expiration of those patents, the ability of other countries to turn to India as the source of alternative supply, and thus the usefulness of compulsory licenses to generate competition and obtain lower prices, may diminish. In this context, compulsory licenses in India and voluntary licenses through organizations such as the Medicines Patent Pool (MPP) may be important in ensuring low-cost, global generic supply for post-1995 drugs.^29-32^ Both compulsory licensing and licensing through the MPP can allow Indian firms to manufacture and export drugs, even when they are covered by primary patents. While compulsory licenses have been rare in India (and not for export), Indian firms are prominent among MPP sub-licensees. The growth of primary patents in India may increase the importance of the MPP going forward.

The key takeaway from this paper is that drugs with primary patent priority year of 1995 or later receive primary patents and face substantially lower levels of generic competition in India. As these drugs represent most new approvals, they are most appropriate for studying the steady-state impact of TRIPS. Though not immediate, India has moved toward the market environment that TRIPS promised to create, one in which new drugs receive strong patent protection that limits competition. This outcome may have important effects on access to medicines globally.

## Supplementary Material

qxaf239_Supplementary_Data

## Data Availability

Public data, analysis code, and replication instructions are available at https://doi.org/10.7910/DVN/5V5UOO. Restricted IQVIA Ark and MIDAS data are subject to commercial agreements and must be obtained directly from IQVIA.
