# Supplementary material for: TRIPS, pharmaceutical patents, and generic competition in India
Source: Health Aff Sch. 2025 Dec 17;4(2):qxaf239. doi: 10.1093/haschl/qxaf239 (PMC12934350; doi:10.1093/haschl/qxaf239)
Supplement: qxaf239_Supplementary_Data [file qxaf239_supplementary_data.zip › Supplemental_Material_Jan2026.pdf]

Supplemental Materials

TRIPS, Pharmaceutical Patents, and Generic Competition in India

Margaret Kyle, Bhaven N Sampat, Kenneth C Shadlen

January 24, 2026

This document contains supplemental materials for:

Margaret Kyle, Bhaven N Sampat, Kenneth C Shadlen, "TRIPS, Pharmaceutical Patents, and Generic Competition in India," *Health Affairs Scholar*, 2025.

https://doi.org/10.1093/haschl/qxaf239

Contents

Appendix 1: Institutional Background and Previous Research 3
Institutional Background 3
Previous Research 3
References for Appendix 1 4

Appendix 2: IQVIA/Ark Patent Categorization 5

Appendix 3: Data Overview 7

Appendix 4: Summary Statistics, Regression Analyses, and Robustness Checks 8
A. Summary Statistics 8
B. Regression Results 10
C. Alternative Lags 12
D. Alternative Measures of Disease Burden 14
E. Non-exact Matches to Indian Drugs 15
F. OLS Regressions of Independent Entry on PPPYear 20

Appendix 1: Institutional Background and Previous Research

Institutional Background

India took several steps in introducing its new pharmaceutical patent system that provide context for and help motivate our analyses.

First, though the WTO and TRIPS came into existence in 1995, developing countries that did not already allow for pharmaceutical product patents were allowed until 2005 to put this new requirement into force. India took full advantage of this transition period: product patent applications were not examined until 2005. As required by TRIPS, all such applications filed after 1995 were held in a "mailbox" until this time, though, as explained in the text, applications with first global filing ("priority") dates before 1995 were ineligible.

India's patent law also includes provisions for "compulsory licenses," which allow for the state to authorize patented inventions to be used without the consent of the patent owner.[1,2] While compulsory license provisions are common features in patent regimes, old and new, India also introduced a novel feature, Section 11(A)7, that essentially allows for automatic compulsory licensing of granted patents based on applications that were deposited in the mailbox. Specifically, Indian generic firms that had made significant investments before 2005 in producing and marketing a drug that later received a patent could continue doing so without being vulnerable to infringement proceedings, subject to payment of a "reasonable royalty."

The most distinctive aspect of Indian TRIPS implementation is Section 3(d) of the Patent Act, which aimed to restrict grants of certain secondary patents. New forms of known substances without a demonstrated improvement in efficacy should not qualify for patents in India under 3(d).[3–5] This provision received global attention when the Indian Patent Office used it to reject a secondary patent filed in 1998 on the leukemia medicine imatinib (Gleevec), a rejection that was ultimately upheld by the Indian Supreme Court.

Previous Research

While economic theory generally predicts that patents restrict competition and raise prices, the limited availability of patents in most low- and middle-income countries meant that little direct empirical evidence from such countries was available when TRIPS was introduced in the 1990s. In the absence of such data, concerns that TRIPS would reduce access to medicines were based on generalizations from the U.S. and European markets, case study evidence, or simulation studies.[6–13]

Early empirical research on realized outcomes in India did not identify substantial effects of TRIPS on pharmaceutical competition and drug prices, as explained in the text. Previous researchers suggest that the small effects of pharmaceutical patents reported in their studies may reflect these aspects of India's TRIPS implementation. Berndt and Cockburn[14] suggest that Section 3(d) may result in many drugs not receiving patent protection in India, and thus account for the high rates of genericization that they observe. Duggan et al.[15] point to compulsory licensing and Section 11(A)7 as potential factors; they also suggest that price controls (which are

separate from TRIPS) may be blunting the effects of patents. These studies do not attempt to estimate the effects of these provisions.

In our assessment, the direct effects of the suggested mechanisms seem limited. Section 3(d) was, if anything, underutilized during the periods covered by prior samples.[16,17] Only one compulsory license has been issued in India since TRIPS, and as far as we could determine Section 11(A)7 has not been invoked. Few of the drugs in previously studied samples were actually subject to price controls, given that these regulations historically focused on non-patented molecules.

It is possible provisions of India's pharmaceutical patent system have indirect effects, e.g. by affecting originators' behavior or encouraging more aggressive launches "at risk" by generic firms, but the most logical explanation for these earlier studies' findings is that they focused on drugs not fully covered by TRIPS. Because of India's strict adherence to the 1995 cut-off date allowed for by TRIPS, drugs with primary patents that have priority dates before 1995 (like imatinib: primary patent priority date April 3, 1992) could only obtain secondary patents, as these could be filed after 1995 and were thus eligible in India.[18,19] Yet not only are applications for secondary patents more vulnerable to rejections, based on Section 3(d) and conventional patentability criteria (e.g. novelty and inventive step), but, even when granted, secondary patents may be easier to invent around and more susceptible to invalidity challenges. Thus, as we explain in the text, the key factor in determining whether a drug is likely to benefit from strong patent protection in India is whether the priority date of its *primary* patent is before or after 1995. This is why we explicitly focus on PPPYear, verifying its effects on the likelihood of getting a primary patent, and, as a result, its effects on generic competition.

References for Appendix 1

1. Reichman J. Compulsory licensing of patented pharmaceutical inventions: evaluating the options. *J Law Med Ethics J Am Soc Law Med Ethics*. 2009;37(2):247-263.

2. Scherer FM, Watal J. Post-TRIPS Options for Access to Patented Medicines in Developing Nations. *J Int Econ Law*. 2002;5(4):913-939.

3. Kapczynski A. Harmonization and its Discontents: A Case Study of TRIPS Implementation in India's Pharmaceutical Sector. *Calif Law Rev*. 2009;97:1571-1649.

4. Gopakumar KM. The Need to Curb Patents on Known Substances. *Econ Polit Wkly*. 2013;48(32):55-57.

5. Basheer S. Trumping TRIPS: Indian patent proficiency and the evolution of an evergreening enigma. *Oxf Univ Commonw Law J*. 2018;18(1):16-45.

6. Nogués JJ. Social Costs and Benefits of Introducing Patent Protection for Pharmaceutical Drugs in Developing Countries. *Dev Econ*. 1993;31(1):24-53.

7. Lanjouw JO. *The Introduction of Pharmaceutical Product Patents in India: Heartless Exploitation of the Poor and Suffering?* National Bureau of Economic Research; 1998.

8. Watal J. Pharmaceutical patents, prices and welfare losses: Policy options for India under the WTO TRIPS agreement. *World Econ*. 2000;23(5):733-752.

9. World Health Organization. *Globalization, TRIPS and Access to Pharmaceuticals*. World Health Organization; 2001.

10. Lanjouw JO. Intellectual Property and the Availability of Pharmaceuticals in Poor Countries. *Innov Policy Econ*. 2003;3:91-129.

11. Chaudhuri S. *The WTO and India's Pharmaceuticals Industry: Patent Protection, TRIPS, and Developing Countries*. Oxford University Press; 2005.

12. Chaudhuri S, Goldberg PK, Gia P. Estimating the Effects of Global Patent Protection in Pharmaceuticals: A Case Study of Quinolones in India. *Am Econ Rev*. 2006;96(5):1477-1514.

13. Goldberg PK. Intellectual Property Rights Protection in Developing Countries: The Case of Pharmaceuticals. *J Eur Econ Assoc*. 2010;8(2-3):326-353.

14. Berndt ER, Cockburn IM. The Hidden Cost Of Low Prices: Limited Access To New Drugs In India. *Health Aff (Millwood)*. 2014;33(9):1567-1575.

15. Duggan M, Garthwaite C, Goyal A. The Market Impacts of Pharmaceutical Product Patents in Developing Countries: Evidence from India. *Am Econ Rev*. 2016;106(1):99-135.

16. Sampat BN, Shadlen KC. Secondary pharmaceutical patenting: A global perspective. *Res Policy*. 2017;46(3):693-707.

17. Ali F, Rajagopal S, Raman VS, John R. Pharmaceutical Patent Grants in India: How our safeguards against evergreening have failed, and why the system must be reformed. 2018.

18. Basheer S. "Prioritising" Pharmaceutical Patents in India? SpicyIP. July 28, 2010.

19. Sampat BN, Shadlen KC. Drug patenting in India: looking back and looking forward. *Nat Rev Drug Discov*. 2015;14:519-520.

Appendix 2: IQVIA/Ark Patent Categorization

The table below shows the patent categorization scheme used by IQVIA/Ark Patent Intelligence.

IQVIA/Ark Patent Categories

**Category**	**Subcategory**
Molecule	Molecule patent
	Salts, hydrates and solvates
	Polymorphic forms
	Other molecule forms
Process and preparation	Intermediates and preparations thereof
	Final synthetic stages
	Complete synthesis
	Purification methods
	Fermentation methods
	Biotechnology
Formulation	General formulation and methods
	Route specific (injectable, oral, ophthalmic, otic, nasal, inhalation, topical, transdermal patch, rectal, vaginal, penile, urinary)
	Kits and packaging
	Excipients
Use	New use related to main indication
	Dosage regimen/administration conditions
	Drug with device
Combination	Novel combination
	Use of combination
Assay	Assay methods
	Patient suitability
Device	Injection
	Respiratory
	Ophthalmic
	Diagnostic
	Energy dependent
	Oral admin

Appendix 3: Data Overview

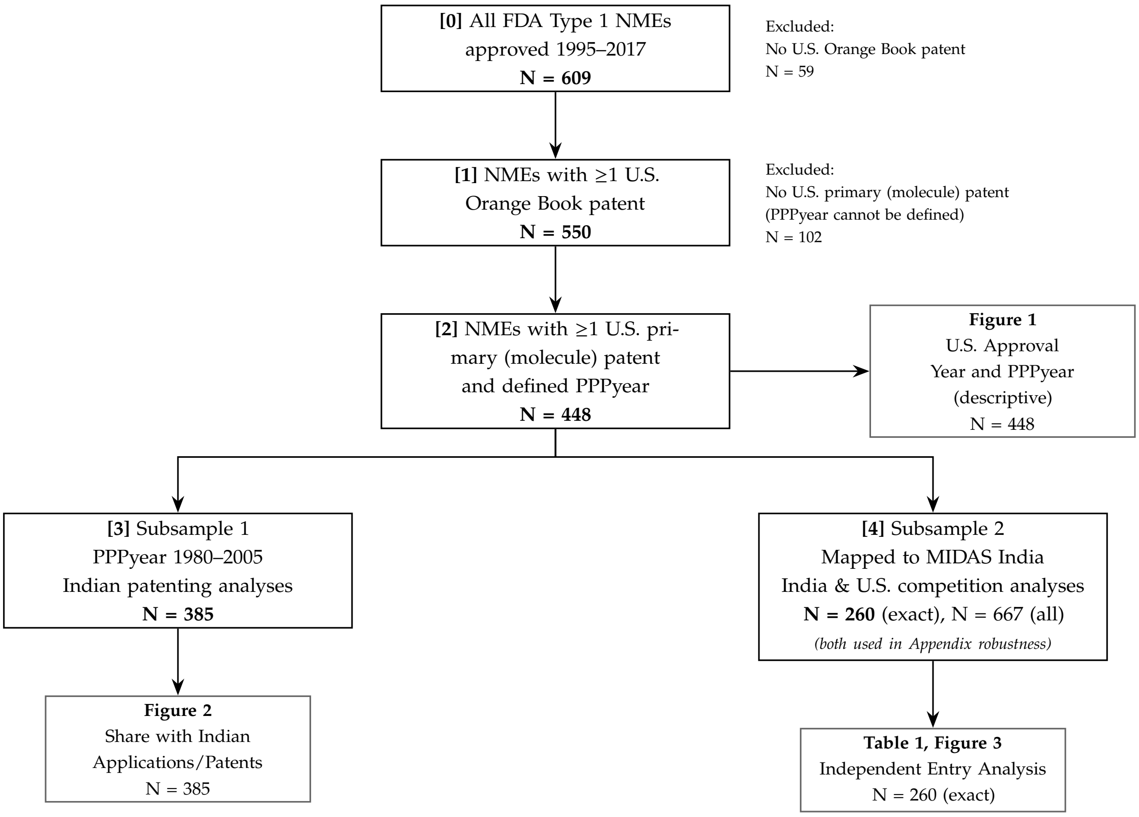

Figure A.1: Sample construction flow diagram showing the progression from all FDA-approved NMEs to the analysis samples. Numbers indicate sample sizes at each step of the data pipeline.

Appendix 4: Summary Statistics, Regression Analyses, and Robustness Checks

A. Summary Statistics

To proxy for drug quality, we used information from Drugs@FDA on whether the drug was approved via the FDA's priority review pathway. Drugs that obtained priority review are those that were expected to represent "significant improvements in the safety or effectiveness of the treatment, diagnosis, or prevention." We also collected other information from Drugs@FDA, including submission type and whether a drug was discontinued in the U.S.

We use the National Library of Medicine's RXNORM database to link drugs to diseases they may treat or prevent. We also use RXNORM to determine the number of U.S.-approved drugs with the same mechanism of action, such as "Protein Kinase Inhibitors" or "RNA Replicase Inhibitors." We collected FDA labels from OpenFDA, from which we determined the number of contraindications and the number of drug interactions for each drug. We use this information as additional proxies for a drug's quality or usefulness, beyond the priority review indicator. This is a proxy of novelty as well as potential experience manufacturers may have with producing similar molecules.

To control for the potential demand in India, we map each drug based on its associated diseases and ICD10 codes from RXNORM to the causes listed in the Institute for Health Metrics and Evaluation's Global Burden of Disease (GBD) data. Table A.1 shows summary statistics for the 260 drugs launched in India that exactly match our U.S. patented NMEs.

Table A.1: Summary statistics, exact NME matches

Variable	Mean	Std. Dev.	Min.	Max.	N
Any independent, 1 year post launch	0.75	0.44	0	1	260
Any independent, 3 years post launch	0.8	0.4	0	1	260
Any independent, 5 years post launch	0.87	0.33	0	1	260
Number of independent producers, 1 year post launch	2.18	2.82	0	19	260
Number of independent producers, 3 years post launch	3.68	4.49	0	22	260
Number of independent producers, 5 years post launch	5.10	6.4	0	36	260
Any Indian application	0.75	0.43	0	1	260
Any Indian patent	0.54	0.5	0	1	260
Any Indian primary patent	0.22	0.41	0	1	260
Any live Indian patent, 1 year post launch	0.48	0.5	0	1	260
Any live Indian patent, 3 years post launch	0.47	0.5	0	1	260
Any live Indian patent, 5 years post launch	0.44	0.5	0	1	260
Any live Indian primary patent, 1 year post launch	0.18	0.39	0	1	260
Any live Indian primary patent, 3 years post launch	0.17	0.38	0	1	260
Any live Indian primary patent, 5 years post launch	0.15	0.35	0	1	260
PPPyear 1995 or later	0.3	0.46	0	1	260
Number of drugs in ATC3, 1 year post launch	13.33	15.06	0	125	260
Number of drugs in ATC3, 3 years post launch	15	16.6	0	151	260
Number of drugs in ATC3, 5 years post launch	16.67	17.79	0	160	260
Average age of other products in ATC3, 1 year post launch	10.2	5.22	0.5	27.45	240
Average age of other products in ATC3, 3 years post launch	10.66	5.48	0.33	29.45	249
Average age of other products in ATC3, 5 years post launch	11.55	5.61	0.84	31.45	251
FDA Priority	0.4	0.49	0	1	260
Oral route of administration	0.72	0.45	0	1	260
Reclassified as BLA	0.02	0.12	0	1	260
Type 1 submission	0.97	0.16	0	1	260
Discontinued in USA	0.24	0.43	0	1	260
Combination product	0	0.06	0	1	260
N drugs with same MOA	60.93	205.23	1	2545	257
N contraindications	3.08	4.78	0	36	260
N associated diseases	2.2	2.27	0	16	260
Log Prevalence (mean)	15.51	2.61	8.44	20	243
Log Prevalence (max)	15.79	2.69	8.73	20	243
Log Prevalence (sum)	15.9	2.74	8.73	20.02	243
Log Years of life lost (mean)	14.11	1.55	8.98	17.42	207
Log Years of life lost (max)	14.35	1.69	8.98	17.42	207
Log Years of life lost (sum)	14.47	1.78	8.98	17.91	207

B. Regression Results

Our main specification considers two measures of independent entry, the market outcome of interest, five years after a drug's launch in India: entry by any independent firm, and the total number of independent producers. Although the dependent variables are discrete, we use a simple linear model due to our relatively small sample size, and because addressing endogeneity is easier than in a non-linear model. Due to missing values for some control variables, the number of observations used in the regressions is 251 in columns controlling for ATC, or 235 in columns with the full suite of controls.

The first two columns of Table A.2 show that overall, drugs with unexpired Indian patents five years after launch are about 15–19 percentage points less likely to have independent generic competition at that time than other drugs in the same ATC class, vintage (U.S. approval year), and with similar characteristics. However, columns 3 and 4 show that drugs with primary patents have a more than 30 percentage point lower likelihood of generic entry than unpatented drugs. Secondary patents are also associated with less generic entry, but the magnitudes are smaller and statistically insignificant. Columns 5–8 show the same specification using the (log) number of independent producers in India, a measure of the extent of competition. Patents overall have a negative (and sometimes statistically insignificant) relationship with competition, but primary patents have a stronger (and consistently significant) negative relationship. The estimates on secondary patents are positive, but insignificant.

Table A.2: OLS regression of independent entry, live patent, launch + 5 years, exact NME matches

	(1)	(2)	(3)	(4)	(5)	(6)	(7)	(8)
	Any	Any	Any	Any	Log(N)	Log(N)	Log(N)	Log(N)
Any live Indian patent, 5 years post launch	-0.145*	-0.187*			-0.159	-0.300*		
	(0.04)	(0.05)			(0.12)	(0.13)		
Any live Indian primary patent, 5 years post launch			-0.312*	-0.330*			-0.867*	-0.854*
			(0.07)	(0.07)			(0.19)	(0.19)
Any live Indian secondary patent, 5 year post launch			-0.046	-0.073			0.088	0.000
			(0.04)	(0.05)			(0.12)	(0.13)
Log number of drugs in ATC3, 5 years post launch	0.077*	0.055*	0.074*	0.057*	0.165*	0.077	0.162*	0.084
	(0.03)	(0.03)	(0.03)	(0.03)	(0.07)	(0.08)	(0.07)	(0.07)
Average age of other products in ATC3, 5 years post launch	0.004	0.008	0.000	0.003	0.019	0.035*	0.006	0.021
	(0.00)	(0.00)	(0.00)	(0.00)	(0.01)	(0.01)	(0.01)	(0.01)
FDA Priority		-0.078		-0.083		-0.162		-0.166
		(0.05)		(0.05)		(0.15)		(0.15)
Oral route of administration		0.099		0.076		0.658*		0.603*
		(0.06)		(0.06)		(0.17)		(0.17)
Reclassified as BLA		-0.527*		-0.569*		-0.740		-0.883+
		(0.20)		(0.19)		(0.55)		(0.53)
Type 1 submission		-0.137		-0.189		-0.036		-0.132
		(0.16)		(0.16)		(0.46)		(0.44)
Discontinued in USA		0.011		0.022		-0.081		-0.054
		(0.05)		(0.05)		(0.14)		(0.14)
Combination product		0.093		0.041		0.423		0.338
		(0.36)		(0.35)		(1.01)		(0.97)
Log N drugs with same MOA		-0.021		-0.028+		-0.002		-0.020
		(0.02)		(0.02)		(0.04)		(0.04)
Log N contraindications		-0.073*		-0.076*		-0.157		-0.167+
		(0.04)		(0.04)		(0.10)		(0.10)
Log N associated diseases		0.051		0.024		0.372*		0.309*
		(0.05)		(0.05)		(0.13)		(0.13)
Log Prevalence (mean)		0.001		0.004		-0.030		-0.025
		(0.01)		(0.01)		(0.04)		(0.03)
Constant	0.672*	1.019*	0.719*	1.139*	1.302*	1.603+	1.486*	1.916*
	(0.13)	(0.32)	(0.13)	(0.32)	(0.36)	(0.90)	(0.35)	(0.88)
R-sqr	0.257	0.339	0.290	0.367	0.228	0.340	0.290	0.387
Obs	251	235	251	235	251	235	251	235

+ and * indicate significance at the 10% and 5% levels, respectively. Specifications include fixed effects for ATC1 and U.S. approval year.

C. Alternative Lags

The main specifications reported above consider the effect of having a live patent granted in India for drug on independent generic competition at five years after local launch. Tables A.3 and A.4 show OLS regressions when measuring generic competition at years one and three, respectively. The results are consistent with those discussed in the main text.

Table A.3: OLS regression of independent entry, live patent, launch + 1 years, exact NME matches

	(1) Any	(2) Any	(3) Any	(4) Any	(5) Log(N)	(6) Log(N)	(7) Log(N)	(8) Log(N)
Any live Indian patent, 1 year post launch	-0.224*	-0.308*			-0.263*	-0.410*		
	(0.06)	(0.06)			(0.10)	(0.10)		
Any live Indian primary patent, 1 year post launch			-0.510*	-0.505*			-0.802*	-0.749*
			(0.08)	(0.08)			(0.14)	(0.13)
Any live Indian secondary patent, 1 year post launch			-0.051	-0.107+			0.010	-0.096
			(0.06)	(0.06)			(0.10)	(0.10)
Log number of drugs in ATC3, 1 year post launch	0.044	0.013	0.047	0.029	0.049	-0.010	0.053	0.013
	(0.04)	(0.04)	(0.03)	(0.04)	(0.06)	(0.06)	(0.06)	(0.06)
Average age of other products in ATC3, 1 year post launch	0.002	0.010	-0.003	0.004	0.012	0.029*	0.001	0.019+
	(0.01)	(0.01)	(0.01)	(0.01)	(0.01)	(0.01)	(0.01)	(0.01)
FDA Priority		-0.070		-0.086		-0.040		-0.060
		(0.07)		(0.07)		(0.12)		(0.11)
Oral route of administration		0.305*		0.237*		0.679*		0.575*
		(0.08)		(0.08)		(0.14)		(0.13)
Reclassified as BLA		-0.162		-0.285		0.144		-0.058
		(0.25)		(0.24)		(0.42)		(0.40)
Type 1 submission		0.086		0.047		0.290		0.244
		(0.21)		(0.20)		(0.35)		(0.33)
Discontinued in USA		0.082		0.104		-0.071		-0.039
		(0.07)		(0.07)		(0.11)		(0.11)
Combination product		0.326		0.291		0.399		0.362
		(0.46)		(0.44)		(0.76)		(0.73)
Log N drugs with same MOA		-0.000		-0.015		0.021		-0.002
		(0.02)		(0.02)		(0.03)		(0.03)
Log N contraindications		0.012		0.002		-0.053		-0.068
		(0.05)		(0.04)		(0.08)		(0.07)
Log N associated diseases		0.104		0.049		0.180+		0.099
		(0.06)		(0.06)		(0.11)		(0.10)
Log Prevalence (mean)		-0.025		-0.020		-0.061*		-0.054*
		(0.02)		(0.02)		(0.03)		(0.03)
Constant	0.770*	0.961*	0.823*	1.099*	0.767*	1.103	0.865*	1.302+
	(0.17)	(0.42)	(0.16)	(0.40)	(0.28)	(0.69)	(0.27)	(0.67)
R-sqr	0.279	0.398	0.362	0.454	0.242	0.390	0.335	0.443
Obs	240	224	240	224	240	224	240	224

+ and * indicate significance at the 10% and 5% levels, respectively. Specifications include fixed effects for ATC1 and U.S. approval year.

Table A.4: OLS regression of independent entry, live patent, launch + 3 years, exact NME matches

	(1) Any	(2) Any	(3) Any	(4) Any	(5) Log(N)	(6) Log(N)	(7) Log(N)	(8) Log(N)
Any live Indian patent, 3 years post launch	-0.181*	-0.232*			-0.208+	-0.336*		
	(0.05)	(0.06)			(0.12)	(0.12)		
Any live Indian primary patent, 3 years post launch			-0.490*	-0.485*			-0.941*	-0.906*
			(0.07)	(0.07)			(0.17)	(0.17)
Any live Indian secondary patent, 3 year post launch			-0.026	-0.066			0.060	-0.027
			(0.05)	(0.05)			(0.11)	(0.12)
Log number of drugs in ATC3, 3 years post launch	0.071*	0.038	0.071*	0.045	0.133+	0.046	0.135*	0.059
	(0.03)	(0.03)	(0.03)	(0.03)	(0.07)	(0.07)	(0.07)	(0.07)
Average age of other products in ATC3, 3 years post launch	0.004	0.008	-0.002	0.002	0.013	0.030*	0.000	0.017
	(0.01)	(0.01)	(0.01)	(0.01)	(0.01)	(0.01)	(0.01)	(0.01)
FDA Priority		-0.075		-0.085		-0.136		-0.148
		(0.06)		(0.06)		(0.14)		(0.14)
Oral route of administration		0.167*		0.138+		0.687*		0.634*
		(0.08)		(0.07)		(0.17)		(0.16)
Reclassified as BLA		-0.324		-0.399+		-0.342		-0.499
		(0.23)		(0.22)		(0.51)		(0.49)
Type 1 submission		-0.102		-0.146		0.294		0.232
		(0.19)		(0.18)		(0.43)		(0.41)
Discontinued in USA		0.079		0.102+		-0.011		0.028
		(0.06)		(0.06)		(0.14)		(0.13)
Combination product		0.091		0.082		0.801		0.816
		(0.43)		(0.40)		(0.95)		(0.90)
Log N drugs with same MOA		0.008		-0.005		0.036		0.012
		(0.02)		(0.02)		(0.04)		(0.04)
Log N contraindications		-0.008		-0.033		-0.052		-0.101
		(0.04)		(0.04)		(0.09)		(0.09)
Log N associated diseases		0.102+		0.064		0.369*		0.303*
		(0.06)		(0.05)		(0.13)		(0.12)
Log Prevalence (mean)		-0.031*		-0.025+		-0.073*		-0.062+
		(0.02)		(0.01)		(0.03)		(0.03)
Constant	0.678*	1.286*	0.739*	1.393*	1.083*	1.473+	1.231*	1.663*
	(0.15)	(0.38)	(0.14)	(0.36)	(0.33)	(0.84)	(0.31)	(0.80)
R-sqr	0.292	0.386	0.384	0.462	0.237	0.358	0.328	0.426
Obs	249	233	249	233	249	233	249	233

+ and * indicate significance at the 10% and 5% levels, respectively. Specifications include fixed effects for ATC1 and U.S. approval year.

D. Alternative Measures of Disease Burden

We also explored different measures of disease burden. Two candidate measures are years of life lost due to a cause (YLL) and the prevalence of a disease. A drug can be mapped to multiple diseases, so we experimented with using the average of the burden measures across all diseases to which a drug is mapped; the maximum; and the sum. While the coefficients on these different measures vary, the coefficients on the patent variables of interest are generally similar.

E. Non-exact Matches to Indian Drugs

In our baseline analyses, we focused on exact matches between the US-approved molecules and products in India because our data on the patent status is most complete for these products. However, many of these molecules are also sold in combination with other molecules in India. For example, the U.S. NME for acarbose has an exact match in India, but this molecule is also sold in combination with another drug (metformin), which is not in our U.S. NME sample.

We undertook the same analyses using the sample of Indian products that included the U.S. molecules in combination with other ingredients as well. Table A.5 shows summary statistics for this broader sample of Indian drugs (all NME matches), and Tables A.6–A.8 regression results (again, at launch +5, +1, and +3 years). In general, the results are again consistent with those presented above.

Table A.5: Summary statistics, all NME matches

Variable	Mean	Std. Dev.	Min.	Max.	N
Any independent, 1 year post launch	0.88	0.33	0	1	667
Any independent, 3 years post launch	0.91	0.29	0	1	667
Any independent, 5 years post launch	0.93	0.25	0	1	667
Number of independent producers, 1 year post launch	2.21	3.43	0	46	667
Number of independent producers, 3 years post launch	3.79	5.82	0	72	667
Number of independent producers, 5 years post launch	5.26	8.38	0	72	667
Any Indian application	0.74	0.44	0	1	667
Any Indian patent	0.56	0.5	0	1	667
Any Indian primary patent	0.13	0.33	0	1	667
Any live Indian patent, 1 year post launch	0.46	0.5	0	1	667
Any live Indian patent, 3 years post launch	0.45	0.5	0	1	667
Any live Indian patent, 5 years post launch	0.42	0.49	0	1	667
Any live Indian primary patent, 1 year post launch	0.09	0.29	0	1	667
Any live Indian primary patent, 3 years post launch	0.08	0.28	0	1	667
Any live Indian primary patent, 5 years post launch	0.07	0.25	0	1	667
PPPyear 1995 or later	0.19	0.39	0	1	667
Number of drugs in ATC3, 1 year post launch	33.11	55.72	0	881	667
Number of drugs in ATC3, 3 years post launch	36.53	58.92	0	933	667
Number of drugs in ATC3, 5 years post launch	39.26	59.82	0	933	667
Average age of other products in ATC3, 1 year post launch	9.85	4.76	0.08	27.45	644
Average age of other products in ATC3, 3 years post launch	10.58	4.98	0.33	29.45	655
Average age of other products in ATC3, 5 years post launch	11.66	5.17	0.84	31.45	657
FDA Priority	0.32	0.47	0	1	667
Oral route of administration	0.79	0.4	0	1	667
Reclassified as BLA	0.02	0.13	0	1	667
Type 1 submission	0.98	0.14	0	1	667
Discontinued in USA	0.29	0.46	0	1	667
Combination product	0.61	0.49	0	1	667
N drugs with same MOA	66.29	241.24	1	2545	662
N contraindications	2.93	4.2	0	36	667
N associated diseases	2.87	2.69	0	16	667
Log Prevalence (mean)	16.24	2.06	8.44	20	636
Log Prevalence (max)	16.69	2.16	8.73	20	636
Log Prevalence (sum)	16.84	2.24	8.73	20.02	636
Log Years of life lost (mean)	14.31	1.43	8.98	17.42	575
Log Years of life lost (max)	14.68	1.63	8.98	17.42	575
Log Years of life lost (sum)	14.85	1.73	8.98	17.91	575

Table A.6: OLS regression of independent entry, live patent, launch + 5 years, all NME matches

	(1)	(2)	(3)	(4)	(5)	(6)	(7)	(8)
	Any	Any	Any	Any	Log(N)	Log(N)	Log(N)	Log(N)
Any live Indian patent, 5 years post launch	-0.079*	-0.111*			-0.032	-0.129		
	(0.02)	(0.02)			(0.09)	(0.09)		
Any live Indian primary patent, 5 years post launch			-0.296*	-0.295*			-0.805*	-0.918*
			(0.04)	(0.04)			(0.16)	(0.16)
Any live Indian secondary patent, 5 year post launch			-0.026	-0.051*			0.090	0.027
			(0.02)	(0.02)			(0.08)	(0.09)
Log number of drugs in ATC3, 5 years post launch	0.034*	0.010	0.032*	0.012	-0.052	-0.090*	-0.056	-0.081+
	(0.01)	(0.01)	(0.01)	(0.01)	(0.04)	(0.04)	(0.04)	(0.04)
Average age of other products in ATC3, 5 years post launch	0.002	0.003	-0.001	0.001	-0.004	-0.001	-0.012	-0.009
	(0.00)	(0.00)	(0.00)	(0.00)	(0.01)	(0.01)	(0.01)	(0.01)
FDA Priority		-0.033		-0.030		-0.294*		-0.285*
		(0.03)		(0.03)		(0.10)		(0.10)
Oral route of administration		0.031		0.032		0.345*		0.363*
		(0.03)		(0.03)		(0.13)		(0.13)
Reclassified as BLA		-0.547*		-0.529*		-1.074*		-1.017*
		(0.09)		(0.08)		(0.34)		(0.34)
Type 1 submission		-0.199*		-0.200*		-0.306		-0.298
		(0.08)		(0.08)		(0.33)		(0.33)
Discontinued in USA		0.020		0.022		-0.151		-0.140
		(0.02)		(0.02)		(0.10)		(0.09)
Combination product		0.044+		0.026		-0.200*		-0.263*
		(0.02)		(0.02)		(0.10)		(0.09)
Log N drugs with same MOA		0.003		-0.004		0.010		-0.011
		(0.01)		(0.01)		(0.03)		(0.03)
Log N contraindications		-0.061*		-0.069*		-0.137+		-0.168*
		(0.02)		(0.02)		(0.08)		(0.08)
Log N associated diseases		0.011		0.001		0.108		0.086
		(0.02)		(0.02)		(0.09)		(0.09)
Log Prevalence (mean)		0.008		0.007		0.009		0.009
		(0.01)		(0.01)		(0.03)		(0.03)
Constant	0.743*	0.953*	0.785*	1.023*	1.460*	1.790*	1.598*	2.002*
	(0.05)	(0.16)	(0.05)	(0.16)	(0.20)	(0.66)	(0.20)	(0.64)
R-sqr	0.180	0.262	0.226	0.300	0.098	0.157	0.133	0.198
Obs	657	627	657	627	657	627	657	627

+ and * indicate significance at the 10% and 5% levels, respectively. Specifications include fixed effects for ATC1 and U.S. approval year.

Table A.7: OLS regression of independent entry, live patent, launch + 1 years, all NME matches

	(1) Any	(2) Any	(3) Any	(4) Any	(5) Log(N)	(6) Log(N)	(7) Log(N)	(8) Log(N)
Any live Indian patent, 1 year post launch	-0.098*	-0.159*			-0.137*	-0.229*		
	(0.03)	(0.03)			(0.06)	(0.06)		
Any live Indian primary patent, 1 year post launch			-0.488*	-0.446*			-0.640*	-0.633*
			(0.05)	(0.05)			(0.10)	(0.10)
Any live Indian secondary patent, 1 year post launch			-0.008	-0.052+			-0.029	-0.096
			(0.03)	(0.03)			(0.06)	(0.06)
Log number of drugs in ATC3, 1 year post launch	0.030*	-0.015	0.029*	-0.006	-0.018	-0.064*	-0.019	-0.052+
	(0.01)	(0.01)	(0.01)	(0.01)	(0.02)	(0.03)	(0.02)	(0.03)
Average age of other products in ATC3, 1 year post launch	0.004	0.008*	-0.001	0.003	0.008	0.014*	0.002	0.008
	(0.00)	(0.00)	(0.00)	(0.00)	(0.01)	(0.01)	(0.01)	(0.01)
FDA Priority		-0.039		-0.034		-0.142*		-0.137*
		(0.03)		(0.03)		(0.07)		(0.07)
Oral route of administration		0.160*		0.140*		0.340*		0.315*
		(0.04)		(0.04)		(0.09)		(0.08)
Reclassified as BLA		-0.309*		-0.245*		-0.264		-0.165
		(0.11)		(0.10)		(0.23)		(0.22)
Type 1 submission		-0.060		-0.007		0.113		0.188
		(0.10)		(0.10)		(0.22)		(0.21)
Discontinued in USA		0.047		0.052+		-0.068		-0.060
		(0.03)		(0.03)		(0.06)		(0.06)
Combination product		0.131*		0.101*		-0.022		-0.064
		(0.03)		(0.03)		(0.06)		(0.06)
Log N drugs with same MOA		0.022*		0.011		0.031		0.015
		(0.01)		(0.01)		(0.02)		(0.02)
Log N contraindications		0.006		-0.001		-0.074		-0.085+
		(0.02)		(0.02)		(0.05)		(0.05)
Log N associated diseases		0.039		0.013		0.047		0.012
		(0.03)		(0.03)		(0.06)		(0.06)
Log Prevalence (mean)		-0.008		-0.006		-0.025		-0.022
		(0.01)		(0.01)		(0.02)		(0.02)
Constant	0.680*	0.709*	0.757*	0.749*	0.761*	0.989*	0.862*	1.034*
	(0.06)	(0.21)	(0.06)	(0.20)	(0.13)	(0.44)	(0.13)	(0.43)
R-sqr	0.232	0.326	0.334	0.392	0.147	0.202	0.194	0.239
Obs	644	614	644	614	644	614	644	614

+ and * indicate significance at the 10% and 5% levels, respectively. Specifications include fixed effects for ATC1 and U.S. approval year.

Table A.8: OLS regression of independent entry, live patent, launch + 3 years, all NME matches

	(1)	(2)	(3)	(4)	(5)	(6)	(7)	(8)
	Any	Any	Any	Any	Log(N)	Log(N)	Log(N)	Log(N)
Any live Indian patent, 3 years post launch	-0.095*	-0.133*			-0.110	-0.191*		
	(0.03)	(0.03)			(0.08)	(0.08)		
Any live Indian primary patent, 3 years post launch			-0.455*	-0.424*			-0.884*	-0.931*
			(0.04)	(0.04)			(0.13)	(0.14)
Any live Indian secondary patent, 3 year post launch			-0.013	-0.043+			0.041	-0.011
			(0.02)	(0.03)			(0.08)	(0.08)
Log number of drugs in ATC3, 3 years post launch	0.033*	-0.007	0.029*	-0.003	-0.046	-0.097*	-0.053	-0.089*
	(0.01)	(0.01)	(0.01)	(0.01)	(0.03)	(0.04)	(0.03)	(0.04)
Average age of other products in ATC3, 3 years post launch	0.003	0.006*	-0.001	0.002	0.003	0.008	-0.006	-0.001
	(0.00)	(0.00)	(0.00)	(0.00)	(0.01)	(0.01)	(0.01)	(0.01)
FDA Priority		-0.043		-0.040		-0.242*		-0.235*
		(0.03)		(0.03)		(0.09)		(0.09)
Oral route of administration		0.083*		0.083*		0.373*		0.378*
		(0.04)		(0.04)		(0.11)		(0.11)
Reclassified as BLA		-0.435*		-0.401*		-0.684*		-0.614*
		(0.10)		(0.10)		(0.31)		(0.30)
Type 1 submission		-0.205*		-0.168+		-0.154		-0.063
		(0.10)		(0.09)		(0.29)		(0.29)
Discontinued in USA		0.028		0.035		-0.121		-0.104
		(0.03)		(0.03)		(0.09)		(0.08)
Combination product		0.098*		0.069*		-0.107		-0.171*
		(0.03)		(0.03)		(0.09)		(0.08)
Log N drugs with same MOA		0.022*		0.011		0.035		0.012
		(0.01)		(0.01)		(0.03)		(0.03)
Log N contraindications		-0.007		-0.024		-0.070		-0.110+
		(0.02)		(0.02)		(0.07)		(0.07)
Log N associated diseases		0.039		0.022		0.109		0.076
		(0.03)		(0.02)		(0.08)		(0.08)
Log Prevalence (mean)		-0.012		-0.011		-0.014		-0.010
		(0.01)		(0.01)		(0.02)		(0.02)
Constant	0.700*	1.071*	0.770*	1.117*	1.156*	1.489*	1.302*	1.575*
	(0.06)	(0.19)	(0.06)	(0.18)	(0.17)	(0.58)	(0.17)	(0.56)
R-sqr	0.223	0.307	0.323	0.381	0.122	0.173	0.179	0.228
Obs	655	625	655	625	655	625	655	625

+ and * indicate significance at the 10% and 5% levels, respectively. Specifications include fixed effects for ATC1 and U.S. approval year.

F. OLS Regressions of Independent Entry on PPPYear

As robustness tests for Figure 3, we estimated the likelihood of independent generic entry as a function of whether a drug's PPPYear is 1995 or later. We did this for both exact matches (Table A.9) and all matches (Table A.10). The results are consistent with Figure 3 and as reported in the text.

Table A.9: OLS regression of independent entry on PPPyear, launch + 5 years, exact NME matches

	(1)	(2)	(3)	(4)
	Any	Any	Log(N)	Log(N)
PPPyear 1995 or later	-0.148*	-0.280*	-0.469*	-0.647*
	(0.04)	(0.07)	(0.12)	(0.20)
Log number of drugs in ATC3, 5 years post launch		0.054+		0.076
		(0.03)		(0.08)
Average age of other products in ATC3, 5 years post launch		0.006		0.030*
		(0.00)		(0.01)
FDA Priority		-0.074		-0.152
		(0.05)		(0.15)
Oral route of administration		0.104		0.696*
		(0.06)		(0.17)
Reclassified as BLA		-0.677*		-1.007+
		(0.20)		(0.53)
Type 1 submission		-0.078		0.095
		(0.17)		(0.45)
Discontinued in USA		0.006		-0.089
		(0.05)		(0.14)
Combination product		0.387		1.118
		(0.37)		(1.02)
Log N drugs with same MOA		-0.028+		-0.018
		(0.02)		(0.04)
Log N contraindications		-0.066+		-0.159
		(0.04)		(0.10)
Log N associated diseases		0.017		0.307*
		(0.05)		(0.13)
Log Prevalence (mean)		-0.001		-0.033
		(0.01)		(0.04)
Constant	0.918*	1.018*	1.533*	1.612+
	(0.02)	(0.33)	(0.06)	(0.89)
R-sqr	0.042	0.331	0.058	0.356
Obs	260	235	260	235

+ and * indicate significance at the 10% and 5% levels, respectively. Specifications include fixed effects for ATC1 and U.S. approval year.

Table A.10: OLS regression of independent entry on PPPyear, launch + 5 years, all NME matches

	(1)	(2)	(3)	(4)
	Any	Any	Log(N)	Log(N)
PPPyear 1995 or later	-0.137*	-0.133*	-0.231*	-0.310*
	(0.02)	(0.04)	(0.09)	(0.15)
Log number of drugs in ATC3, 5 years post launch		0.012		-0.085+
		(0.01)		(0.04)
Average age of other products in ATC3, 5 years post launch		0.004+		-0.002
		(0.00)		(0.01)
FDA Priority		-0.020		-0.280*
		(0.03)		(0.10)
Oral route of administration		0.028		0.361*
		(0.03)		(0.13)
Reclassified as BLA		-0.545*		-1.034*
		(0.09)		(0.34)
Type 1 submission		-0.154+		-0.220
		(0.08)		(0.33)
Discontinued in USA		0.010		-0.163+
		(0.02)		(0.10)
Combination product		0.047+		-0.192*
		(0.02)		(0.10)
Log N drugs with same MOA		0.001		0.007
		(0.01)		(0.03)
Log N contraindications		-0.050*		-0.127+
		(0.02)		(0.08)
Log N associated diseases		-0.021		0.056
		(0.02)		(0.09)
Log Prevalence (mean)		0.005		0.007
		(0.01)		(0.03)
Constant	0.959*	0.952*	1.402*	1.762*
	(0.01)	(0.17)	(0.04)	(0.65)
R-sqr	0.046	0.248	0.010	0.160
Obs	667	627	667	627

+ and * indicate significance at the 10% and 5% levels, respectively. Specifications include fixed effects for ATC1 and U.S. approval year.
